# Predictors of self-care behaviors and glycemic control among patients with type 2 diabetes mellitus

**DOI:** 10.3389/fpubh.2022.1031655

**Published:** 2023-01-11

**Authors:** Towhid Babazadeh, Yosef Lotfi, Soheila Ranjbaran

**Affiliations:** ^1^Department of Public Health, Sarab Faculty of Medical Sciences, Sarab, Iran; ^2^Department of Nursing, Sarab Branch, Islamic Azad University, Sarab, Iran

**Keywords:** self-care, type 2 diabetes, extended theory of reasoned action, HbA1c, Iran

## Abstract

**Aims:**

This study used the Extended Theory of Reasoned Action (ETRA) to predict self-care behaviors and HbA1c among patients with type 2 diabetes in Iran.

**Materials and methods:**

A cross-sectional study was performed using a multistage random sample. A total of 240 patients with type 2 diabetes, who were referred to the diabetes healthcare centers in Chaldoran, participated in the research. Instruments consisting of standardized questionnaires were used based on the Extended Theory of Reasoned Action (ETRA) constructs and the summary scale of diabetes self-care behaviors measure.

**Findings:**

The results of this study demonstrated that demographic variables explained ~ 7% (*p*-value = 0.23) and ETRA constructs 18% of the variance (*p*-value = 0.02) in behavioral intention, respectively. According to the hierarchical multiple linear regressions on self-care behaviors, demographic factors (*p*-value 0.001) dictated 45.7% of the variation of the self-care behavior, while knowledge, attitude, self-efficacy, and behavioral intention (*p*-value 0.001) accounted for 63.4% of the variance. The ETRA constructs, self-care practices, and demographic factors together account for almost 57% of the variation in the HbA1c. Self-care practices were the best indicator of HbA1c (β = −0.593).

**Conclusion:**

ETRA constructs and self-care behavior can be the best determinants of HbA1c level in type 2 diabetes. This model is suggested to be applied in designing intervention programs to improve HbA1c in these groups of patients.

## Introduction

Diabetes is a chronic, metabolic disease, which leads to heart attacks, and blood vessel, eye, kidney, and nerve problems (1). According to the report of the World Health Organization (WHO), “In 2019, diabetes was the ninth leading cause of death with an estimated 1.5 million deaths directly caused by diabetes” (2). People with type 2 diabetes involve more than 95% of individuals with diabetes (2). In Iran, the prevalence of chronic type 2 diabetes has grown from 5.7% in 2010 to 14.3% in 2019 (3, 4). To reduce and prevent complications of diabetes, patients with diabetes need good self-care behaviors (5).

Individuals', families', and communities' ability to promote health, prevent disease, retain health, and cope with illness and disability with or without a health worker's support is self-care (6). Self-care behaviors in patients with type 2 diabetes focus on a healthy diet, regular exercise, blood sugar testing, medication adherence, foot care, and smoking cessation (7, 8). Evidence indicates that the main reason for the failure of diabetes health programs is the lack of attention given to self-care behaviors in patients with diabetes (9, 10). Self-care behaviors lead to useful outcomes, including promoted health and quality of life; increased patient satisfaction; decreased health costs; better symptom management; increased life expectancy; controlling the progression of type 2 diabetes; and prevention of serious complications such as cardiovascular risk factors, decreased hospitalization rates, and improved glycemic control (11–15). HbA1c level is one of the most important indicators of glycemic control in patients with type 2 diabetes, which can be affected by various factors such as social, clinical, and psychological issues (16).

Despite the importance of self-care behaviors and glycemic control among patients with diabetes, only 15.1% of Iranian patients with type 2 diabetes adhere well to self-care behaviors (17). Therefore, it seems necessary to identify the determinants of self-care behaviors and HbA1c in these groups of patients.

The current study used the Extended Theory of Reasoned Action (ETRA) to predict self-care behaviors and HbA1c among patients with type 2 diabetes. The previous studies showed that using behavior change theories and models, such as ETRA (TRA with self-efficacy), successfully determined healthy behavior factors (18, 19). The study by Tavousi et al. demonstrated that ETRA constructs predicted 36% of intention and 28% of substance abuse behavior variance in adolescents (20). The Theory of Reasoned Action (TRA), developed by Ajzen and Fishbein, is one of the best theories for behavioral change (21). In this model, individual behaviors are affected by the attitude toward a certain action and its outcome, subjective norms, intentions, and behaviors (21, 22). ETRA was created using TRA constructs along with the self-efficacy construct of Social Learning Theory (SLT). TRA has been extended by the addition of a construct that includes self-efficacy (20, 23).

Self-efficacy determines behavioral intentions as the third factor, plus attitude and subjective norm (24). Also, self-efficacy could directly associate with behavior (23, 24). That is to say, the importance of self-efficacy in predicting health behaviors. Experience with mastery is an important factor in improving self-efficacy (25). The experience of mastery is necessary for decision-making in implementing self-care behaviors among patients with type 2 diabetes. Hence, the present study aimed to use the Extended Theory of Reasoned Action (ETRA) in predicting self-care behaviors and HbA1c among patients with type 2 diabetes. One of the strengths and novelties of this study was predicting self-care behavior and HbA1c among patients with diabetes in rural areas.

## Methods

### Study design and participants

We applied a cross-sectional study design to use the Extended Theory of Reasoned Action (ETRA) in predicting self-care behaviors and HbA1c among patients with type 2 diabetes who were referred to the diabetes healthcare centers in Chaldoran, Iran, from May to July 2019.

Based on a previous study (26), the sample size using the G^*^Power software was calculated at 240 patients. Multistage random sampling was employed to recruit 240 cases. Four comprehensive health centers were randomly selected out of eight, and the cases at the four centers entered the study based on their records. The sampling method was as follows: Electronic health records entitled SIB (an abbreviation for the Persian equivalent of “integrated health system”) exist for all Iranian citizens within the SIB system, so from the central server, we could extract all the cases of type 2 diabetes followed by the Health Centers of Chaldoran city.

In this way, from each rural center and according to the diabetic population of that center, samples were selected randomly using MS Excel software, with the command “=Randbetween (bottom, top)” selecting samples.

Inclusion criteria for this study included patients diagnosed with type 2 diabetes, consenting to participate in the study, and being within the age range of 18–65 years. Exclusion criteria included patients with gestational diabetes, ulcers on the feet, and progressive cardiovascular conditions.

### Ethical considerations

The study protocol was reviewed and approved by the ethics committee of Tabriz University of Medical Sciences (ethical code: 93183). Written informed consent was obtained from all participants after fully explaining the nature, purpose, and procedures used for the study. For illiterate participants, *via* a face-to-face interview, all questionnaires were completed by an interviewer. Respondents were also assured that their information would remain confidential.

## Measures

### Demographic information questionnaire

Demographic information included age, gender, job, education level, disease duration, marital status, and household monthly income.

### Self-care behaviors questionnaire

To assess self-care behaviors (such as observing the diet, physical exercise, regularly consuming diabetic medications, and self-monitoring of blood glucose), we used the summary scale of diabetes self-care behaviors measure developed by Tolbert et al. (27), which had been validated and whose reliability was verified by Didarloo et al. in Iran (19).

### ETRA-based questionnaire

The data collection instrument was a valid and reliable ETRA-based questionnaire and a knowledge scale (19). The ETRA questionnaire included four constructs: attitude (4 items), subjective norms (4 items), self-efficacy (4 items), and behavioral intention (2 items). For all four constructs, the items were rated on a 5-point Likert-type scale ranging from 1 to 5 (1 = complete disagreement through 5 = full agreement). The higher the scores, the more attitude, subjective norms, self-efficacy, and behavioral intention toward conducting self-care behaviors were presented.

The question responses were on a Likert scale, and the response category for each item ranged from 1 = strongly disagree to 5 = strongly agree.

The behavioral attitude was measured using the following statements: “I think that following a special diet plan makes to delay the complications related to diabetes” and “delaying diabetes complications by following a diet program is fully important for me”. For measurement of subjective norms of self-care behaviors: “My family supports me to participate in a suitable exercise” and “My family's opinion always is important for me”.

Self-efficacy was evaluated using the following statements: “I think I am able to follow my diet when I am away from home”, “I am able to take my medicines as prescribed”, and “I am able to check my blood sugar if necessary”.

The behavioral intention of the people with diabetes was measured using the following statements: “I intend to take all my diabetes medications exactly as prescribed during the future month” and “I plan to self-monitor my blood sugar once a week during the future month”.

Knowledge of self-care behaviors among people with diabetes was measured using 11 multiple-choice items. An example is “What are the early complications of diabetes?”. For “don't know” responses one score, for mistake responses 0; and for correct responses, two scores were allocated. So, the total possible score for each questionnaire was 0 to 22.

### Glycemic control

The new HbA1c value was retrieved from the patient's medical record and employed as a glycemic management metric.

### Statistical analysis

Data were analyzed by SPSS software version 20. Continuous variables were summarized as mean (±SD) if they were normally distributed and median (and interquartile range) otherwise. Also, categorical variables were reported as numbers (and percentages). Before comparing variables, the best transformation for each non-normally distributed parameter was assessed by the Shapiro–Wilk test. Continuous variables among groups were examined using a one-way analysis of variance (ANOVA) and independent samples *t*-test. Pearson's correlation coefficient was used to investigate the correlation between ETRA constructs and self-care behaviors.

A hierarchical multiple regression analysis was performed to examine the unique contribution of demographic and ETRA constructs in the explanation of self-care behavior (28). In step 1, behavioral intention was the dependent variable, and (a) demographic variables, (b) knowledge, attitude, subjective norms, and self-efficacy were the independent variables. In step 2, the five subscales of the ETRA were entered into the regression equation, which was used to measure the impact of the ETRA on self-care behaviors.

## Results

In total, 240 patients who met the eligibility criteria were enrolled in the study, with a mean age of 54.40 ± 8.50 years. The surveyed population consisted of 53.3% men and 46.7% women. A significant difference was observed between self-care behaviors and HbA1c with age groups (*p*-value< 0.001); in other words, the lowest self-care and highest HbA1c level among age groups belonged to the age group over 50 years. Also, in terms of behavioral intention, the lowest score was for the age group of 40–49 years (*p*-value = 0.022). The highest self-care score and the lowest HbA1c were among patients with an education level of guidance school and higher rather than illiterate or elementary school (*p*-value < 0.05). There was a significant increase in self-care behaviors with an increase in the disease duration, that is, 6–10 years and 10 years (*p*-value< 0.001). As well, the HbA1c level in patients with a history of long-term disease was significantly lower than in other patients (*p*-value< 0.05). Additional data are summarized in Table 1.

**Table 1:** Demographic characteristics and their associations with self-care behaviors among diabetic patients.

**Variables**	**Subgroups**	**N (%)**	**Self-care behaviors**		**Behavioral intention**		**HbA1c**	
			**Mean (**±**SD)**	***p*** **-value**	**Mean (**±**SD)**	***p*** **-value**	**Mean** ±**SD**	***p*** **-value**
Age groups^*^	30–39	32 (13.3)	18.7 (**±**3.2)	0.001	24.0 (**±**4.1)	0.022	6.62 (±0.95)	0.001
	40–49	58 (24.2)	15.2 (**±**3.8)		20.3 (**±**4.1)		7.07 (±0.99)	
	≥50	150 (62.5)	13.5 (**±**2.8)		20.6 (**±**4.8)		7.79 (±0.88)	
Gender^**^	Male	128 (53.3)	14.6 (**±**3.3)	0.880	20.5 (**±**50.7)	0.241	6.84 (±1.03)	0.380
	Female	112 (46.7)	14.5 (**±**3.8)		21.5 (**±**4.3)		6.96 (±1.04)	
Job^**^	Employed	142 (59.2)	15.7 (**±**2.2)	0.529	20.9 (**±**4.8)	0.746	6.82 (±1.07)	0.139
	Unemployed	98 (40.8)	14.5 (**±**3.6)		21.7 (**±**1.8)		7.02 (±0.98)	
Level of education^*^	Illiterate	84 (35.0)	13.2 (**±**2.6)	0.002	20.3 (**±**3.9)	0.537	7.21 (±1.09)	0.002
	Elementary	76 (31.7)	14.6 (**±**3.4)		21.1 (**±**5.3)		6.85 (±1.07)	
	Guidance school and higher	80 (33.3)	16.0 (**±**4.1)		21.5 (**±**5.0)		6.65 (±0.88)	
Duration of disease (year)^*^	5 ≤	56 (23.3)	12.7 (**±**2.9)	0.001	20.5 (**±**4.8)	0.513	7.75 (±0.94)	0.001
	6–10	126 (52.5)	14.0 (**±**2.5)		20.7 (**±**4.8)		6.73 (±0.90)	
	≥10	58 (24.2)	17.7 (**±**4.2)		21.8 (**±**4.4)		6.45 (±0.95)	
Marital status^**^	Single	32 (13.3)	14.8 (**±**2.0)	0.768	21.3 (**±**6.1)	0.730	6.85 (±1.01)	0.751
	Married	208 (86.7)	14.5 (**±**3.7)		20.9 (**±**4.5)		6.91 (±1.04)	
Household monthly income^*^	>307 dollars	30 (12.5)	16.9 (**±**4.3)	0.001	22.7 (**±**4.6)	0.081	7.42 (±0.70)	0.001
	307–460 dollar	160 (66.7)	14.3 (**±**3.0)		20.7 (**±**4.6)		6.88 (±0.99)	
	>460 dollars	50 (20.8)	12.2 (**±**3.1)		19.5 (**±**4.8)		7.42 (±1.04)	

* *P*-value calculated based on one-way ANOVA.

** *P*-value calculated based on *t*-test.

Using Pearson's correlation coefficient, it was found that HbA1c level had a statistically significant positive correlation with knowledge (*r* = 0.563), attitude (*r* = 0.404), self-efficacy (*r* = 0.348), behavioral intention (*r* = 0.365), and self-care behaviors (*r* = 0.731), while subjective norms had no significant association (*r* = −0.083) (Table 2).

**Table 2:** Bivariate correlations of ETRA variables and self-care behaviors among diabetic patients.

**Variables**	**1**	**2**	**3**	**4**	**5**	**6**	**7**
1 = Knowledge	1						
2 = Attitude	0.237^*^	1					
3 = Subjective norms	0.064	0.091	1				
4 = Self-efficacy	0.088	0.005	0.020	1			
5 = Behavioral intention	0.219^*^	0.144^*^	0.198^*^	0.265^*^	1		
6 = Self- care behaviors	0.586^**^	0.368^**^	0.083	0.305^**^	0.242^*^	1	
7 = HbA1c	0.422^*^	0.276^*^	0.045	0.341^*^	0.263^*^	0.731^*^	1

* Correlation is significant at the 0.05 level (2-tailed).

** Correlate is significant at the 0.01 level (2-tailed).

As shown in Table 3, demographic variables explained ~ 7% of the variance of behavioral intention, which was not statistically significant at 0.05 levels. However, ETRA constructs were responsible for an 18% change in observed variance, which was statistically significant (*p*-value = 0.02). The other hierarchical multiple linear regressions were conducted with self-care behaviors (Table 4), and a significant effect was observed on self-care behaviors by demographic characteristics in the first block (R^2^ = 0.457, *p*-value < 0.001); also, in the second block, knowledge, attitude, and self-efficacy, as well as behavioral intention, were significant positive predictors of self-care behaviors (R^2^ = 0.634, *p*-value < 0.001). Knowledge, attitude, behavioral intention, and self-efficacy were the strongest predictors of self-care behaviors, respectively.

**Table 3:** Hierarchical regression analysis to predict the behavioral intention among diabetic patients.

**Step/variable**	B (Step 1)^*^	*p*-value^**^	B (Step 2)^*^	*p*–value^**^
(1) Age	−0.15	0.070	−0.10	0.258
Gender	0.11	0.052	0.08	0.107
Level of education	0.03	0.543	0.02	0.849
Job	0.01	0.722	0.02	0.978
Marital status	0.08	0.239	0.01	0.884
Duration of disease	0.04	0.380	0.03	0.891
Income status	0.13	0.078	0.05	0.440
(2) Knowledge			0.15	0.014
Attitude			0.06	0.676
Subjective norms			0.22^*^	0.002
Self-efficacy			0.20^*^	0.001
R^2^	0.07		0.18	

* The unstandardized regression coefficients (B).

** *p* < 0.05.

**Table 4:** Hierarchical regression analysis to predict self-care behaviors among diabetic patients.

**Step/variable**	B (Step 1)^*^	*p*-value^**^	B (Step 2)^*^	*p*-value^**^
(1) Age	−0.325	0.001	−0.211	0.001
Gender	0.010	0.959	0.110	0.013
Level of education	0.141	0.029	0.062	0.432
Job	0.078	0.015	0.006	0.768
Marital status	0.161	0.014	0.059	0.228
Duration of disease	0.365	0.001	0.225	0.001
Income status^*^	0.210	0.001	0.122	0.001
(2) Knowledge			0.303	0.001
Attitude			0.188	0.001
Subjective norms			0.012	0.364
Self-efficacy			0.163	0.001
Behavioral intention			0.142	0.021
R^2^	0.457		0.634	

* The unstandardized regression coefficients (B).

** *p* < 0.05.

To predict HbA1c, we employed the Hierarchical Multiple Linear Regression (Table 5). In step 1, demographic characteristics were significant predictors of HbA1c, as shown in Table 4 (*P*-value = 0.001, R2 total = 0.334). Age, disease duration, and income status were the strongest predictors of self-care HbA1c, respectively. After including the ETRA constructs and self-care behaviors in the model (step 2), both behavioral intention (*p* < 0.001) and self-care behaviors (*p* < 0.001) were significant predictors of HbA1c with R2 = 0.575. This means that the ETRA constructs, self-care behaviors, and demographic variables account for about 57.5% of the variation in the HbA1c. Self-care behaviors were the strongest predictor of HbA1c (ß = −0.593).

**Table 5:** Hierarchical linear regression to predict HbA1c among diabetic patients.

**Step/variable**	β^**^(Step 1)	*p*-value^*^	β^**^(Step 2)	*p*-value^*^
(1) Age groups	−0.272	0.001	−0.072	0.169
Gender	0.074	0.178	0.064	0.175
Level of education	0.055	0.330	0.031	0.504
Job	0.036	0.533	0.016	0.741
Marital status	0.096	0.095	0.002	0.974
Duration of disease	−0.333	0.001	0.071	0.191
Income status	0.202	0.001	0.049	0.326
(2) Knowledge			−0.002	0.974
Attitude			0.003	0.955
Subjective norms			−0.003	0.955
Self-efficacy			−0.093	0.493
Behavioral intention			−0.241	0.031
Self-care behaviors			−0.593	0.001
R^2^	0.334		0.575	

* *p* < 0.05.

** β is a standardized coefficient.

## Discussion

One of the main elements of a care plan in patients with diabetes is to consider an appropriate self-care and glycemic control plan for them, and an important precondition for self-management is self-efficacy which plays a serious role in diabetes patients' self-care. Therefore, this study was carried out to use the Extended Theory of Reasoned Action (ETRA) in predicting self-care behaviors and HbA1c among patients with type 2 diabetes.

A significant difference was observed among self-care behaviors, HbA1c level, and aging. Also, there was a relationship between behavioral intention and age groups. The highest self-care score and lowest HbA1c level were among patients with an education level of guidance school and higher rather than illiterate or elementary school. There was a significant increase in self-care behaviors with increasing disease duration. As well, the HbA1c level was significantly lower in patients with a history of long-term disease than in other patients. As the income level of households increased, it was noted that self-care behaviors were significantly reduced and HbA1c levels were increased. Most of the previous studies reported significant differences among self-care behaviors, HbA1c status, and socio-demographic factors among patients with type 2 diabetes (29–37). A study in Northern Jordan demonstrated that HbA1c level is significantly related to household income, education levels, employment status, and disease duration (16). Due to the nature of chronic diabetes, aging can associate with increased disability, forgetfulness, fatigue, increased complications, and the severity of the disease, which can lead to a reduction in self-care behaviors and an increased HbA1c level in patients with diabetes. Additionally, as the duration of the disease increases, patients are more likely to cope better with their diseases and gain the knowledge and skills to overcome the barriers and promote self-care behaviors that lead to a decrease in HbA1c.

Also, patients with higher levels of education are likely to have better knowledge and are better able to understand and apply self-care behavioral guidelines than those with lower levels of education. The findings of a study in Tanahun, Nepal (31), showed that illiterate patients performed poor self-care behaviors three times more often than literate patients. According to Tiruneh et al. (38), education status positively affects self-care behaviors in patients with type 2 diabetes. Another factor influencing patients' self-care behavior and HbA1c was the level of household income. The effect of income on people's health and health-promoting behaviors is obvious. It can be explained that performing many self-care behaviors in patients with diabetes, including blood glucose control at home, routine laboratory blood testing, diet, and medication adherence, are cost-dependent. As a result, low-income levels and self-care confidence lead to lower self-care maintenance in patients with type 2 diabetes (39). Thus, interventional programs in terms of patients' self-care with diabetes and HbA1c level should focus on specific age groups, household income, and duration of disease, as well as according to the education level of patients.

The self-care behaviors in patients and HbA1c level had a statistically significant positive correlation with knowledge, attitude, self-efficacy, and behavioral intention, while subjective norms had no significant association. Also, self-care behaviors were the strongest predictor of HbA1c. Therefore, the factors that affect the self-care behavior of patients with type 2 diabetes can also affect HbA1c. These findings are consistent with the results of the previous study by Babazadeh et al. (40), who reported that all ETRA constructs were determinants of self-care behaviors in patients with type 2 diabetes except subjective norms. According to another study's results, among structures of ETRA, knowledge, self-efficacy, and behavioral intention were determinants of physical activity behavior in patients with type 2 diabetes (18). The literature has highlighted increasing knowledge, attitude, and behavioral intention along with self-efficacy which leads to increased self-care behaviors in patients with type 2 diabetes (19, 41, 42). It can be explained that none of the factors alone can motivate a person to engage in self-care behaviors, but these factors together lead the person to develop self-care behaviors and control the disease. The current study showed that the most critical determinant of HbA1c was self-care behaviors. Incessantly, some studies found that adherence to self-care behaviors was significantly related to low levels of HbA1c (16, 33, 35). Hence, it seems necessary to focus on identifying the factors affecting self-care behavior in patients with type 2 diabetes based on the appropriate model and designing interventions by healthcare providers aiming to promote self-care behaviors and decrease HbA1c levels in these groups of patients.

In the present study, demographic variables explained ~ 7% of the variance of behavioral intention, which was not statistically significant. However, ETRA constructs were responsible for an 18% change in the observed variance of behavioral intention, which was statistically significant. According to the results of hierarchical regression analysis, demographic characteristics explained 45.7% of the variance of self-care behaviors and 33.4% of the variance of HbA1c, and in the second block, knowledge, attitude, and self-efficacy, as well as behavioral intention, explained 63.4% of the variance of self-care behaviors. Knowledge, attitude, behavioral intention, and self-efficacy were the strongest predictors of self-care behaviors, respectively. Also, both behavioral intention and self-care behaviors were significant predictors of HbA1c (57.5%). The strongest predictor of HbA1c was self-care behaviors.

Babazadeh et al.'s study reported that ETRA constructs described 70% of the variation of behavioral intention and 30% of self-care behaviors in patients with type 2 diabetes (40). A similar study in Iran reported that ETRA constructs explained 41.0% of the behavioral changes in physical activity among patients with type 2 diabetes and self-efficacy was the strongest predictor (18). A study conducted by Didarloo et al. demonstrated that ETRA constructs described 41.5% of the intentions and 25.3% of the behavior variance among women with type 2 diabetes (19). In the study conducted by Almomani et al., the exhibited socio-demographic, clinical, and psychological distress variables, such as BMI, type of treatment, income, education level, and psychological distress, explained 22.3% of the variance in HbA1c levels, and diabetes self-care behaviors (except medication) accounted for 30.3% of the variance in HbA1c levels. Meanwhile, the total variance was 52.6%, and exercise was the most important predictor of HbA1c (16).

In this study, although the model has successfully determined behavioral intention, self-care behavior, and glycemic control, it has probably been more important in determining self-care behavior than intention. Knowledge was the strongest predictor of self-care behaviors. It shows that knowledge plays the main role in health-promoting behaviors, especially self-care in patients with type 2 diabetes. Patients must have knowledge regarding their disease and self-care behaviors to achieve the desired treatment goals and proper management of their diseases (43). Another study indicated that knowledge, attitude, and social support were determinants of self-care behaviors, and knowledge was the strongest predictor of self-care behaviors in patients with type 2 diabetes (41). In studies, the observed diabetes-related knowledge and self-care knowledge were associated with self-care behaviors (38, 42). Patients who have better knowledge about the symptoms of diabetes are more successful in testing their blood sugar and exercising (34, 44). Knowledge, attitudes, and behaviors to promote health-related behaviors and prevent diseases are related (41). Individuals will perform healthy behaviors when they have knowledge about their diseases and the ways of self-care and have a positive attitude toward such behaviors, where this attitude can turn into behavioral intention (45, 46). Behavioral intention is required to perform the actual behavior but is not a guarantee of performing the behavior alone (46). Also, self-efficacy has a critical role in improving self-care to prevent complications in type 2 diabetes, and a higher level of self-efficacy leads to better levels of self-care behaviors (47). Information regarding type 2 diabetes and the patient's self-efficacy for self-care in controlling symptoms and psychological complications are factors in successful diabetes management (47). In this study, it was seen that the HbA1c level was affected by self-care behaviors. Therefore, the factors that affect self-care behaviors are more likely to change HbA1c, which should be considered in this group of patients.

It seems that general education and guideline for all patients cannot lead to self-care goals and improving HbA1c levels. It is suggested that education of the patients regarding self-care and glycemic control be provided through individual education, focusing on demographic characteristics and considering clinical conditions, knowledge and perceptual abilities, beliefs, and environmental conditions. Meanwhile, using models and theories of behavior change appropriate to the country and the region's population can help identify the factors affecting self-care behaviors and HbA1c in patients.

## Limitations

As the data collection method in the current study was based on self-reports by the patients, recall bias is warranted.

## Conclusion

ETRA constructs were practically supported to identify the potential factors of diabetes self-care behavior and glycemic control. This research highlights the effect of knowledge, attitude, self-efficacy, and behavioral intention on intensifying the level of self-care behavior and HbA1c among patients with type 2 diabetes.

## Data availability statement

The original contributions presented in the study are included in the article/supplementary material, further inquiries can be directed to the corresponding author.

## Ethics statement

The studies involving human participants were reviewed and approved by Ethical Committee of Tabriz University of Medical Sciences. The patients/participants provided their written informed consent to participate in this study.

## Author contributions

SR and TB: designing the study, conducting the study, administrative support, and drafting and revising the manuscript. YL: contributed to the study concept and design, and interpretation of the data. All authors contributed to the article and approved the submitted version.
